# Dual-hemisphere anodal transcranial direct current stimulation improves bilateral motor synergies

**DOI:** 10.3389/fpsyg.2023.1211034

**Published:** 2023-07-20

**Authors:** Hanall Lee, Joon Ho Lee, Tae Lee Lee, Do-Kyung Ko, Nyeonju Kang

**Affiliations:** ^1^Department of Human Movement Science, Incheon National University, Incheon, Republic of Korea; ^2^Neuromechanical Rehabilitation Research Laboratory, Incheon National University, Incheon, Republic of Korea; ^3^Division of Sport Science, Health Promotion Center, Sport Science Institute, Incheon National University, Incheon, Republic of Korea

**Keywords:** bilateral force control, bilateral motor synergy, dual anodal stimulation, tDCS, primary motor cortex (M1)

## Abstract

Transcranial direct current stimulation (tDCS) is one of the non-invasive brain stimulation techniques that can improve motor functions. As bimanual motor actions require high motor cortical activations between hemispheres, applying bilateral anodal stimulation on left and right sides of primary motor cortex (M1) can improve for improvements in bimanual motor tasks. This study investigated which bilateral tDCS protocol effectively improves bimanual hand-grip force control capabilities in healthy young adults. We used three different bilateral tDCS protocols: (a) dual-anodal stimulation on the M1 of bilateral hemispheres (Bi-AA), (b) anodal–cathodal stimulation on the M1 of dominant and nondominant hemispheres (Bi-AC), and (c) sham stimulation (Sham). The results indicated that applying the Bi-AA significantly improved bilateral motor synergies estimated by uncontrolled manifold analysis relative to Sham. However, these differences were not observed in the comparison between Bi-AA and Bi-AC as well as between Bi-AC and Sham. These findings suggest that facilitating motor cortical activations between both hemispheres may be an additional option for advancing interlimb motor coordination patterns.

## 1. Introduction

Transcranial direct current stimulation (tDCS) is a non-invasive brain stimulation technique that may change cortical activations by delivering weak direct electrical direct current via electrodes to the targeted area of the head (Nitsche and Paulus, 2000; Nitsche et al., 2008). The potential mechanisms of tDCS posited that the application of anodal stimulation facilitates neuronal activities with depolarization of somatic membrane potential, whereas cathodal stimulation inhibits neural activities with hyperpolarization of somatic membrane potential (Nitsche and Paulus, 2000). In addition to possible simultaneous effects of tDCS (i.e., online effects), effects of tDCS may last a few hours after the stimulation (i.e., offline effect) (Coppens et al., 2019). Transition from the initial membrane potential may transfer to a longer period of synaptic plasticity modification similar to long-term potentiation and long-term depression via modulation of N-methyl-D-aspartate receptors (Stagg and Nitsche, 2011). In summary, tDCS protocols may modulate brain activation patterns presumably related to various motor and cognitive functions in human.

The application of tDCS may enhance motor performances via facilitation of cortical excitability (Kidgell et al., 2013; Ghasemian-Shirvan et al., 2023; Schwell et al., 2023). Conventional tDCS protocols for motor improvements involve providing anodal stimulation to the primary motor cortex (M1) of the unilateral hemisphere with a return electrode on the contralateral supra orbital area (Nitsche et al., 2009). For healthy young individuals, anodal stimulation on the M1 of dominant hemisphere improved finger sequencing coordination and grip force control within their contralateral hand (Kang and Paik, 2011; Fan et al., 2017). To facilitate motor improvements, recent studies suggested the use of bilateral tDCS protocols that targeted left and right M1 areas, including anodal stimulation on the dominant hemisphere and cathodal stimulation on the nondominant hemisphere (i.e., anodal–cathodal stimulation) (Vines et al., 2008; Williams et al., 2010). As the bilateral tDCS protocol may facilitate cortical activations in the M1 of dominant hemisphere while reducing interhemispheric inhibitions from the nondominant hemisphere (Williams et al., 2010; Lindenberg et al., 2013), this approach has been shown to produce greater unimanual motor improvements than the conventional tDCS protocols in healthy younger adults. It is interesting to note that the bilateral tDCS protocol can be used to improve bimanual motor performances, associated with successful activities of daily living (Furuya et al., 2014). However, the anodal-cathodal stimulation may focus on strengthening motor functions of dominant hand contributing to bimanual motor improvements through asymmetrical polarity effects on each hemisphere (Furuya et al., 2013, 2014). Successful bimanual movements typically require increased motor activations between both hemispheres (Gomes-Osman and Field-Fote, 2013). Thus, an alternative option for bimanual motor improvements may be anodal stimulation on the M1 of bilateral hemispheres (i.e., dual-anodal stimulation). Overall, it is necessary to identify the optimal bilateral tDCS protocol for developing tDCS rehabilitation programs aimed at enhancing bimanual motor functions.

The purpose of this randomized sham-controlled crossover study was to investigate which bilateral tDCS protocol effectively improves bimanual motor functions in healthy young adults. Bilateral tDCS protocols included the following three options: (a) dual-anodal stimulation on the M1 of bilateral hemispheres (Bi-AA), (b) anodal–cathodal stimulation on the M1 of dominant and nondominant hemispheres (Bi-AC), and (c) sham stimulation (Sham). We administered online tDCS protocols to participants while performing bimanual isometric force control tasks, which effectively assessed their bimanual motor functions (Almuklass et al., 2016; Jin et al., 2019). The bimanual force control capabilities were estimated by performance and coordination variables. Bimanual performance variables included force accuracy (root mean square error, RMSE) and variability (coefficient of variation, %CV), and interlimb force coordination was evaluated by quantifying uncontrolled manifold (UCM) variables across multiple trials. Based on the findings of previous studies (Gomes-Osman and Field-Fote, 2013; Hadoush et al., 2018), we hypothesized that dual-anodal stimulation of the M1 of bilateral hemispheres would transiently enhance bimanual force control performances and interlimb coordination in comparison to those for Bi-AC and Sham conditions.

## 2. Methods

### 2.1. Participants

Sixteen healthy young adults with no musculoskeletal impairments (6 females and 10 males; mean ± standard deviation = 24.38 ± 2.53 years; all right-handed) participated in this study. We conducted *a priori* power analysis based on the pilot data using the G*Power software (version 3.1.9.4) and calculated an adequate sample size (Faul et al., 2007). This analysis indicated that at least 16 participants per group were required in a within subject design (power = 0.99 and alpha = 0.05). We directed participants to avoid excessive exercises, physical activities, and alcohol use within 24 h, and consumption of any substances such as caffeine and pain killer within 12 h before the experiment. This study complies with provisions in the Declaration of Helsinki and was performed according to the study protocol approved by the Incheon National University’s Institutional Review Board (No. 7007971–201,904-002A). We confirmed that all participants read and signed a written informed consent prior to the study.

### 2.2. tDCS protocols

**Figure 1:**
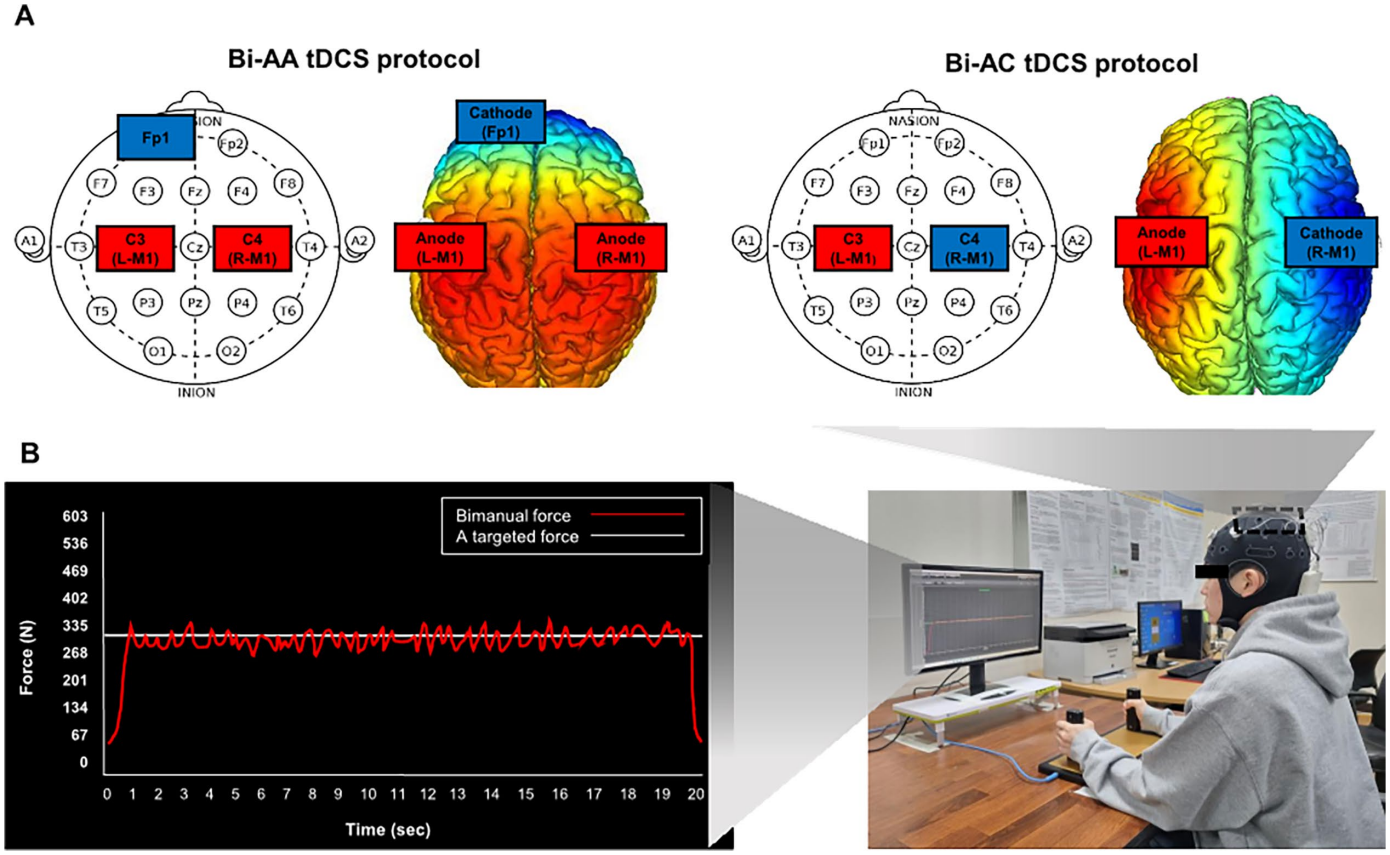
Bimanual force control performances and experimental procedures with bilateral tDCS montage. **(A)** Bilateral tDCS montages and **(B)** bimanual force control tasks using isometric force grip measurement system while receiving two online visual information. A red line denoted bimanual forces, a sum of forces from left and right hands and a white horizontal line in the middle indicated a targeted force level.

All participants underwent each session of Bi-AA, Bi-AC, and sham conditions in a random order. We used a wireless tDCS device (Starstim 8-Neuroelectronics^®^, Barcelona, Spain) that targeted hand regions of M1. Bilateral tDCS protocols were included (Figure 1A): (1) Bi-AA: anodal stimulation on dominant and non-dominant M1 (C3 and C4) with cathodal stimulation on left supraorbital area (Fp1) and (2) Bi-AC: anodal stimulation on the dominant M1 (C3) hemisphere and cathodal stimulation on the non-dominant M1 (C4). For the Sham condition (i.e., dual-sham stimulation on the M1 of bilateral hemisphere with cathodal stimulation on Fp1), participants were subjected to an electrical current of 1.0 mA within the first 30 s, and then it was ramped down without informing them beforehand (Fonteneau et al., 2019). Bilateral tDCS parameters were as follows: (1) intensity = 2.0 mA, (2) electrode size = 25 cm^2^, (3) current density = 0.08 mA/cm^2^, (4) the duration of a session = 20 min, and (5) density charge = 0.096C/cm^2^.

### 2.3. Bimanual isometric force control

We used a customized isometric hand-grip force measurement system for bimanual isometric force control tasks (SEED TECH Co., Ltd., Bucheon, South Korea). The isometric hand-grip force measurement system includes left and right handles (a diameter = 30 mm) with two force transducers embedded on each side (Micro Load Cell-CZL635-3135, range = 220 lbs., Phidgets Inc., Calgary, Canada). The experimental procedures for force control tasks were performed using a customized Microsoft Visual C++ Program (Microsoft Corp., Redmond, United States). Isometric force data were acquired at the 200 Hz of sampling rate using a 16-bit analog-to-digital converter (A/D; ADS1148 16-Bit 2kSPS and a minimum detectable force = 0.0192 N), and the data were additionally amplified using INA122 with an excitation voltage of 5 V (Texas Instruments Inc., Dallas, USA).

### 2.4. Experimental procedures

Participants were seated 80 cm away from a LED monitor (1,920 × 1,080 pixels; a refresh rate = 60 Hz), and requested to sit comfortably and rest their forearms on the table to minimize unintended force production by elbow, shoulder, and trunk movements. During the task, participants bimanually produced hand-grip forces (Figure 1B). Each participant initially underwent two maximum voluntary contraction (MVC) trials (duration = 5 s and rest between trials = 110 s), and we calculated the average value of peak force values from each MVC trial to obtain the individual’s MVC value. For the bimanual isometric force control tasks, we set a targeted force level (i.e., 40% of MVC) for each participant.

The goal of bimanual isometric force control task was to produce and match their bimanual forces (i.e., the sum of left and right forces) to the targeted level for 20 s using two visual information cues (Figure 1B). Each participant completed a total of 10 trials with 110 s of rest time between consecutive trials. Bilateral tDCS was simultaneously provided while performing bimanual isometric force control task. Each participant was randomly assigned one of the three bilateral tDCS protocols, including Bi-AA, Bi-AC, and Sham, and a wash-out period greater than 5 days was allowed to minimize potential carryover effects (Lee and Kang, 2023).

### 2.5. Data analyses

For the following offline analyses, we used a customized Matlab program (Math Works™ Inc., Natick, United States). The force data were preprocessed through a bidirectional fourth-order Butterworth filter with 30 Hz cut off frequency. To minimize early initial adjustment and termination effects, we removed the first 3 s and last 3 s of force data for each trial, and used the middle 14 s of data for analysis. We estimated bimanual force control capabilities by quantifying force control performances and coordination patterns as follows: (a) force accuracy = RMSE, (b) force variability = %CV (SD/mean force × 100), and (c) force asymmetry = left force/right force (e.g., values close to 1 indicate symmetrical forces between hands), and interlimb force coordination (i.e., UCM variables).

**Figure 2:**
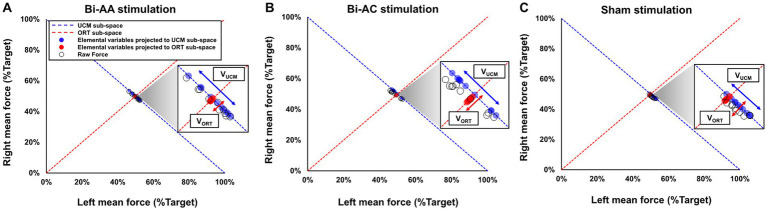
Representative UCM data during bimanual force control across three tDCS protocols. **(A)** Bi-AA stimulation (i.e., bilateral dual-anodal stimulation), **(B)** Bi-AC stimulation (i.e., bilateral anodal-cathodal stimulation), and **(C)** Sham stimulation. UCM analysis focuses on 10 pairs of normalized elemental variables (i.e., raw force produced by left and right hands and represented as black circles) from each trial. Blue circles indicated elemental variables projected to UCM sub-space (i.e., blue dotted line) and red circles denote elemental variables projected to ORT sub-space (i.e., red dotted line orthogonal to blue dotted line). The variance of blue circles (i.e., V_UCM_: good variability) does not change bimanual force control performances whereas the variance of red circles (i.e., V_ORT_: bad variability) interferes with bimanual force control performances.

Consistent with previous UCM findings (Latash, 2010; Kang and Cauraugh, 2017), we quantified bilateral motor synergies. We averaged left and right force outputs from each trial, and normalized the two mean force values relative to the target force level, respectively. Next, we considered the pair of two normalized left and right mean force values as an elemental variable for each trial. For example, when the targeted force level was 100 N, participant could produce 90 N of mean total force from two hands for a trial (i.e., 40 N of left mean force and 50 N of right mean force). Then, a pair of normalized elemental variables equals to (40, 50%): (a) left element variable: 40 N/100 N × 100 = 40% from left hand and (b) right element variable: 50 N/100 N × 100 = 50% from right hand. We performed the same calculation in all 10 trials which enabled us to acquire 10 pairs of elemental variables for each experimental condition. Then, all elemental variables were projected to sub-spaces of UCM and ORT (i.e., sub-space orthogonal to UCM), respectively (Figure 2). Good variability (V_UCM_) is a variance of elemental variables projected to UCM sub-space indicating various motor solutions in an uncontrolled manner (i.e., limitless combinations of left and right forces equal to the target force) although this variance does not affect task performances because the solution equals to the exact amount of force from the task. Bad variability (V_ORT_) is a variance of elemental variables projected to ORT sub-space interfering with stability of task performances across multiple trials. The sum of the two variability components (i.e., V_UCM_ + V_ORT_) is the total variance (V_TOT_). Using Equation 1, we calculated the index of bilateral motor synergies (V_Index_) for each force control trial, and then we performed a Z-transformation to conduct an additional parametric analysis (Equation 2). V_Index_ denotes the proportion of V_UCM_ and V_ORT_ when performing bimanual force control tasks, and a high value of V_Index_ indicates a high task stability across multiple trials.


(1) $$V_{Index}=\frac{V_{UCM}∕df_{UCM}-V_{ORT}∕df_{ORT}}{V_{TOT}∕df_{TOT}}$$

(2) $$V_{Index}\left(Z-transformed\right)=0.5\times \ln\frac{2+V_{\mathrm{Index}}}{2-V_{\mathrm{Index}}}$$

where the degrees of freedom for good variability (df_UCM_) and bad variability (df_ORT_) is 1 and the degrees of freedom for total variability (df_TOT_) is 2. Consistent with the UCM hypothesis the values of V_Index_ ranged from − 2 to 2. Figure 2 shows examples of good and bad variability from representative data for each bilateral tDCS protocol: (a) Bi-AA (Figure 2A), (b) Bi-AC (Figure 2B), and (c) Sham (Figure 2C).

To check data for normality, we performed the Shapiro–Wilk’s test, which confirmed that three out of six dependent variables were normally distributed (i.e., RMSE, force symmetry, and V_Index_). For these three dependent variables, we conducted one-way repeated measure ANOVAs to test for statistical significance among the three bilateral tDCS protocols (i.e., Bi-AA, Bi-AC, and Sham). For the remaining three dependent variables (i.e., CV, V_UCM_, and V_ORT_), which were not normally distributed, we conducted the non-parametric Friedman test. Bonferroni’s pair-wise comparisons were conducted for the post-hoc analysis. We used the IBM SPSS Statistics 25 (SPSS Inc., Chicago, IL, United States) to conduct all the statistical analyses and the alpha level was set at 0.05.

## 3. Results

**Figure 3:**
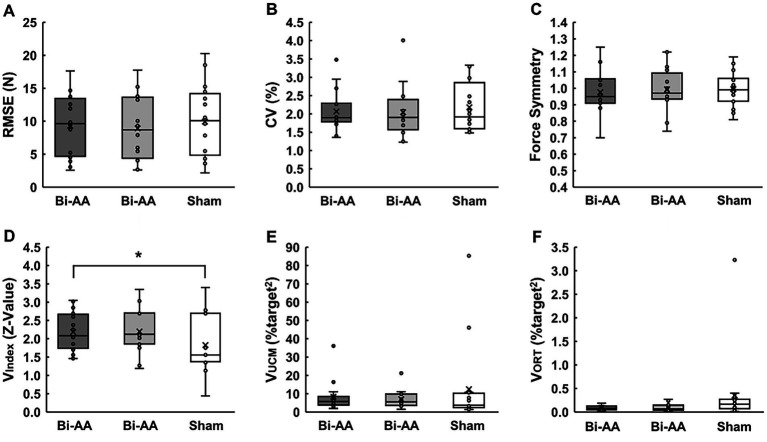
Bimanual force control performances across three tDCS protocols. **(A)** Force accuracy, **(B)** force variability, **(C)** force symmetry, **(D)** bilateral motor synergies, **(E)** good variability, and **(F)** bad variability. Box plot show individual data (black circles), mean (X sign in the box), median (black horizontal line in the box), interquartile range (IQR = *Q*3-*Q*1; top and bottom of the box indicates *Q*3, and *Q*1), maximum value: *Q*1 + 1.5 × IQR, and minimum value: *Q*1–1.5 × IQR. Asterisk (*) denotes a significant difference between the Bi-AA and Sham tDCS protocol.

One-way repeated measure ANOVAs and the Friedman test on bimanual force control performance variables showed no significant difference among the following three bilateral tDCS protocols: (a) RMSE: *F*_2, 30_ = 1.277; *p* = 0.294; η^2^ = 0.078 (Figure 3A), (b) %CV: χ^2^(2) = 0.875; *p* = 0.646 (Figure 3B), and (c) Force Asymmetry: *F*_2, 30_ = 0.625; *p* = 0.542; η^2^ = 0.040 (Figure 3C). Interestingly, one-way repeated measure ANOVAs on UCM variables found a significant difference with respect to bilateral motor synergies (V_Index_): *F*_2, 30_ = 4.570; *p* = 0.019; η^2^ = 0.234 (Figure 3D). The post-hoc analysis showed that values of V_Index_ for: (a) the Bi-AA were significantly greater than those for the Sham (*p* = 0.025). No statistical difference was found in the comparison between Bi-AA and Bi-AC (*p* > 0.999), as well as between Bi-AC and Sham (*p* = 0.126). Additionally, the Friedman test failed to report significant differences among three bilateral tDCS protocols: (a) good variability (V_UCM_): χ^2^(2) = 0.500; *p* = 0.779 (Figure 3E) and (b) bad variability (V_ORT_): χ^2^(2) = 3.500; *p* = 0.174 (Figure 3F). These results suggest that a session of dual-anodal stimulation on the M1 of bilateral hemispheres transiently improved motor synergies between left and right hand-grip forces across multiple trials.

## 4. Discussion

This study investigated which bilateral tDCS protocol effectively improves bimanual hand-grip force control capabilities in healthy young adults by focusing on three different bilateral tDCS protocols including Bi-AA, Bi-AC, and Sham. Bilateral motor synergies estimated by uncontrolled manifold analysis were significantly higher in the Bi-AA condition than those in sham stimulation, whereas this difference was not observed in the comparison between Bi-AA and Bi-AC as well as between Bi-AC and Sham. Bimanual force control performances were not significantly different among the three bilateral tDCS protocols.

Importantly, we found that applying dual-anodal stimulation on the M1 of bilateral hemispheres significantly improved bilateral motor synergies (i.e., greater values of V_Index_) in comparison to sham stimulation condition. According to the UCM theory (Latash, 2010), increased bilateral motor synergies demonstrating advanced interlimb coordination patterns are associated with greater stabilization of task performances across multiple trials. Moreover, achieving such synergetic interlimb motor coordination typically involves higher levels of cognitive functions, such as executive functions, which minimize motor errors between trials (McCombe Waller et al., 2008). The cumulative UCM findings demonstrated that greater bilateral motor synergies were observed in individuals with better fine motor control capabilities (Vaz et al., 2019). Despite no significant difference in bilateral motor synergies between Bi-AA and Bi-AC, our findings indicated the possibility that facilitating motor cortical regions of bilateral hemispheres can improve interlimb force coordination patterns across multiple trials.

Earlier research has shown that bimanual motor tasks involve a high level of activation in the motor cortical regions of both hemispheres (McCombe Waller et al., 2008). However, in healthy individuals, anodal–cathodal stimulation on the M1 of both the dominant and nondominant hemispheres has often been used to enhance dominant hand function, resulting in effective execution of bimanual motor tasks (Williams et al., 2010). Furthermore, patients who had a stroke typically showed impairments in executing and coordinating bimanual motor actions due to imbalanced motor cortical activations between the hemispheres. Interestingly, although a previous bilateral tDCS protocol for stroke motor recovery used anodal stimulation on affected hemisphere and cathodal stimulation on unaffected hemisphere based on the interhemispheric competition model (Di Lazzaro et al., 2014), recent studies that used dual-anodal stimulation for bilateral hemispheres have highlighted the positive role of the ipsilateral pathway from the unaffected hemisphere in the recovery of the paretic arm (Kang et al., 2018). Presumably, bimanual motor functions can be enhanced by facilitating motor cortical activations as well as corticospinal projections from dominant and nondominant hemispheres. In addition to the conventional bilateral tDCS protocol (i.e., Bi-AC), Bi-AA protocol may be an additional option for improving bimanual motor coordination patterns.

Despite bilateral motor synergies being improved by dual-anodal stimulation, we found no significant differences in bimanual force control performances. Previous meta-analytic findings revealed that multiple tDCS sessions applied to the M1 resulted in greater motor improvements during finger tapping, serial reaction time, and isometric pinch force control tasks compared to a single tDCS session (Hashemirad et al., 2016). It is possible that a single session of tDCS may transiently influence motor coordination strategies, but our results suggest that this was insufficient to improve overall task performances during bimanual force control. Future studies should investigate the effects of multiple dual-anodal stimulation sessions on bimanual motor functions. Moreover, given that values of bilateral motor synergies after Bi-AA were not significantly different from those after Bi-AC, further investigations focusing on various bimanual motor tasks are needed to determine which tDCS protocol is an effective option for optimizing bimanual motor improvements.

In conclusion, our study revealed that applying dual-anodal stimulation on M1 of bilateral hemispheres transiently increased bilateral motor synergies estimated by UCM analysis during bimanual isometric force control tasks in relative to those for sham stimulation. However, we did not find a significant difference between Bi-AA and Bi-AC as well as between Bi-AC and Sham, and further bimanual force control performances were not significantly different among the three bilateral tDCS protocols. Thus, we tentatively conclude that enhancing motor cortical activations between hemispheres may be an additional bilateral tDCS protocol for improving interlimb force coordination patterns across multiple trials.

## Data availability statement

The original contributions presented in the study are included in the article/supplementary material, further inquiries can be directed to the corresponding author.

## Ethics statement

The studies involving human participants were reviewed and approved by the Incheon National University’s Institutional Review Board. The patients/participants provided their written informed consent to participate in this study. Written informed consent was obtained from the individual(s) for the publication of any potentially identifiable images or data included in this article.

## Author contributions

NK provided the idea of the manuscript. NK and HL prepared the manuscript draft. NK, HL, JL, TL, and DKK contributed to the experiment design, data collection, and data interpretation. All authors have read and approved the final version of the manuscript.

## Funding

This work was supported by the Ministry of Education of the Republic of Korea and the National Research Foundation of Korea (NRF-2020S1A5A8041203) to NK.

## Conflict of interest

The authors declare that the research was conducted in the absence of any commercial or financial relationships that could be construed as a potential conflict of interest.

## Publisher’s note

All claims expressed in this article are solely those of the authors and do not necessarily represent those of their affiliated organizations, or those of the publisher, the editors and the reviewers. Any product that may be evaluated in this article, or claim that may be made by its manufacturer, is not guaranteed or endorsed by the publisher.
